# ANALYSIS OF FINE MOTOR CONTROL IN INSTITUTIONALIZED SHELTERED CHILDREN AND ADOLESCENTS THROUGH PERFORMANCE IN COMPUTER SOFTWARE

**DOI:** 10.1590/1984-0462/2020/38/2018377

**Published:** 2020-05-08

**Authors:** André Wesley de Araujo, Rafaela Villa Almeida, Tania Brusque Crocetta, Carlos Bandeira de Mello Monteiro, Deborah Cristina Gonçalves Luiz Fernani, Maria Tereza Artero Prado Dantas

**Affiliations:** a Universidade do Oeste Paulista, Presidente Prudente, SP, Brazil.; b Universidade do Estado de Santa Catarina, Florianópolis, SC, Brazil.; c Universidade de São Paulo, São Paulo, SP, Brazil.

**Keywords:** Learning, Institutionalization, Motor skills, Diagnosis, Aprendizagem, Institucionalização, Habilidade motora, Diagnóstico

## Abstract

**Objective:**

To analyze the psychomotor development and the fine motor control of institutionalized and non-institutionalized sheltered children and adolescents.

**Methods:**

A cross-sectional study in which 54 subjects participated and were divided into two groups: 27 institutionalized sheltered children and adolescents (SG) and 27 non-institutionalized sheltered children and adolescents (CG). The psychomotor battery and the Learning and Motor Control software were used to evaluate development and motor control. The analysis of variance was performed for both groups with repetitive measurements for the last factor.

**Results:**

The SG presented a total development score inferior to the CG, with differences in tonicity (p=0.041) and body awareness (p=0.039). The longest distance was performed on Task 1 (M=983.9 pixels; diagonal line; distance of 930.053 pixels), with no difference between the groups (p=0.64). Furthermore, the SG presented a greater average time in Task 1 (M=16.12 seconds) when compared with Tasks 2 (M=11.6 seconds; horizontal line; distance of 750 pixels) and 3 (M=10.6; vertical line; distance of 550 pixels), but only marginally different between Tasks 2 and 3 (p=0.055). Regarding the number of correct answers, the CG scored more (M=6.1) when compared with SG (M=4.6), with p<0.05.

**Conclusions:**

The institutionalized individuals showed a psychomotor development inferior to the CG. Furthermore, they presented impairment in fine motor control, covering a larger distance on the task that required the diagonal movement, longer execution time, less correct answers, and more errors.

## RESUMO

**Objetivo:**

Analisar o desenvolvimento psicomotor e o controle motor fino de crianças e adolescentes institucionalizados e não institucionalizados em abrigo.

**Métodos:**

Estudo transversal, no qual participaram 54 indivíduos, divididos em dois grupos: 27 crianças e adolescentes institucionalizados em abrigo (GA) e 27 crianças e adolescentes não institucionalizados (GC) em abrigo. Para avaliação do desenvolvimento e controle motor, foram utilizadas a bateria psicomotora e o *software* Aprendizagem e Controle Motor. Foi realizada a análise de variância para os dois grupos com medidas repetidas para o último fator.

**Resultados:**

O GA apresentou pontuação total do desenvolvimento inferior ao GC, com diferença na tonicidade (p=0,041) e noção corporal (p=0,039). A maior distância percorrida encontrada foi na Tarefa 1 (M=984,9 *pixels*; com reta diagonal; distância de 930,053 *pixels*), sem diferença entre os grupos (p=0,64). Além disso, o GA apresentou tempo médio da Tarefa 1 (M=16,1 segundos) superior às Tarefas 2 (M=11,6 segundos; reta horizontal; distância de 750 *pixels*) e 3 (M=10,6 segundos; reta vertical; distância de 550 *pixels*), mas apenas marginalmente diferente entre as Tarefas 2 e 3 (p=0,055). Já em relação ao número de acertos, o GC apresentou mais acertos (M=6,1) comparado ao GA (M=4,6), com p<0,05.

**Conclusões:**

Os indivíduos institucionalizados apresentaram desenvolvimento psicomotor inferior ao GC, além de comprometimento no controle motor fino com maior distância percorrida na tarefa que exigia o movimento em diagonal, maior tempo na execução, menos acertos e mais erros.

## INTRODUCTION

In childhood, motor skills are acquired from learning, and at this stage, it is important to provide children with activities that involve movements to promote adequate development and growth. Children, in this period, sharpen their senses, for example, by acquiring greater sensitivity to stimuli from the environment. This will then reflect on their global development until they’re adults.^1^

The family nucleus and the relationships a child maintains with their environment are essential for their development. As a result, these stimuli and lived experiences will influence their motor learning.^2^ Studies show that children living in poorly structured homes are exposed to risky situations, neglect and/or some type of abuse. In addition, many are taken to a host institution.^2^ ^,^ ^3^ These individuals who go through the institutionalization process may have their motor learning affected, as they are exposed to precarious psychosocial care and environment stimuli, which are capable of affecting their development, especially their motor control.^4^

Data on the number of children and adolescents living in shelter institutions are scarce. However, the United Nations Children’s Fund (UNICEF) estimates that at least eight million children live in institutionalized settings around the world. In Brazil, according to the National Register of Children and Adolescents in Shelters (*Cadastro Nacional de Crianças e Adolescentes Acolhidos* - CNCA), created by the National Justice Counsel (Conselho Nacional de Justiça - CNJ), 36,551 children and adolescents were living in shelters in 2011.^5^ ^,^ ^6^

Nevertheless, however welcoming the shelters may be, they do not necessarily offer an adequate environment for a child’s global development, due to the lack of direct contact with a family nucleus and due to the high density of children within a shelter and few caregivers. This fact can cause a deficit of stimuli, quality care, attention and the formation of affective bonds,^7^ which can compromise the control and execution of certain movements, justifying the need for this study.

Fine motor control involves manual skill, guided by vision, in which minimal force and great precision are used to execute a movement. This occurs by activating the sensorimotor cortex area of the hands and fingers, which provides coordination and fine movement. This is because part of motor learning is acquired from the first years of life.^8^ In order to evaluate fine motor control in different populations, several tasks have already been used, such as: functional tasks with geometric objects and shapes (evaluating various aspects such as grip strength, and hand-eye coordination) in individuals with Down’s syndrome. However this is applicable only up to 42 months. Computer softwares have also been used to evaluate precision and agility in individuals with cerebral palsy, but it is not possible to evaluate their coordination and manual dexterity.^9^ ^,^ ^10^

Considering this context, the present study aimed to analyze the motor development and the performance of fine motor control of institutionalized and non-institutionalized children and adolescents.

## METHOD

This was a cross-sectional study (CAAE: 63119716.5.0000.55) carried out in two shelters in the city of Presidente Prudente, in the state of São Paulo, with the participation of 54 individuals, divided into two groups: Sheltered Group (SG), with 27 children and adolescents institutionalized in a shelter, aged between 6 and 18 years old, and of both sexes; Control Group (CG), with 27 non-institutionalized individuals, who lived with a family and were participants in an educational and sports project carried out in the same space as one of the shelters. The individuals in the CG were paired by sex and age with the SG, that is, for each individual assessed in the SG, a subject from the CG was selected, with the same sex and age (with a maximum difference of up to six months). The inclusion criteria for both groups were: institutionalized and non-institutionalized children and adolescents, with authorization to participate in the research as approved by the legal guardian and the individual. In addition, all of the institutionalized individuals in shelters with the aforementioned age range and availability of time for evaluation were included in the SG, whereas individuals in the CG were recruited after the end of the SG evaluations, so as to select those with the same ages and genders of the SG members. The exclusion criteria were: individuals who had physical, visual, hearing impairments or some cognitive and neurological deficit observed by the assessment team and/or reported by the shelter and project coordinators. Seven individuals from the SG were excluded from the study, one with cognitive impairment due to intellectual disability and the other due to the impossibility of evaluation, because of work and study schedules and/or because they were going to leave the shelter to return to live with a family.

The evaluations were carried out in the shelter spaces. The psychomotor battery (PMB),^11^ was applied. It was validated to assess the psychomotor development (PD) of school-age children and adolescents.^12^ It analyzes tonicity, balance, laterality, body awareness, temporal structure, and global and fine praxis. The result from this instrument is obtained with the average of each factor analyzed, and it is possible to classify the individual as: apraxic (7‒8 points); dyspraxic (9‒13 points); normal (14‒21 points); good (22‒26 points) and superior (27‒28 points).^11^

**Figure 1:**
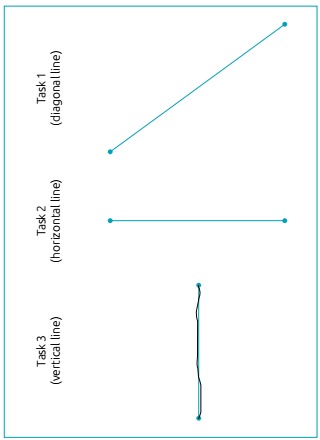
Presentation of the three lines from the tasks used in the study, and the image of the task also shows the path taken by a participant (in black).

To evaluate the fine motor skill of the upper limbs, three tasks were used in the Motor Evaluation and Control (MEC) software,^13^ ^,^ ^14^ ^,^ ^15^ ^,^ ^16^ which is valid and reliable for the evaluation of fine, discrete, and closed motor skill control and thus providing reliable parameters. This instrument was used for the evaluation process of hemiparetic patients^16^ and integrates with the Wacom Intuos Creative Pen & Touch Tablet^®^, in which the participant must follow a straight red line drawn on their computer screen as quickly as possible. The child uses a special pen to draw on the graphics tablet, and the outline made by the child is shown on the computer screen in black. The graphics tablet does not show the line drawn by the participant, as both are presented on the computer screen. This requires the participant to demonstrate visomotor coordination between the hand movement with the pen on the table and the line drawn on the computer screen. The participant cannot remove the pen from contact with the tablet or else the task will be marked as “invalid” (error), and a new attempt must be initiated. The participant starts the task at a point on the line (shown by a red circle) and must proceed to the end point (another red circle). The *software* records the distance of the line, the distance traveled by the participant, the time to complete the task and whether the attempt was successful (correct). ^13^ ^,^ ^14^ ^,^ ^15^ ^,^ ^16^ The experiment consisted of three tasks described below. Each of them was repeated by the participants, in up to ten attempts each. The first task consisted of a diagonal line, with a starting point at the coordinates x, y=50,50 and an end point at coordinates x,y=800,600, with a real distance of 930,053 *pixels*. The second task consisted of a horizontal line (coordinates x,y=50,400 and x,y=800,400) with a real distance of 750 pixels. The third task, on the other hand, consisted of a vertical line (coordinates x,y=400,50 and x,y=400,600) with a real distance of 550 pixels (Figure 1).

For data analysis, we used the average for the distance covered and the time spent in carrying out the tasks that were considered valid (correct answers), in addition to the number of correct answers for each task.

Statistical analysis was performed using the Statistical Package for the Social Sciences software (version 20.0.0, IBM Corporation, Armonk, New York, USA). Analysis of variance (ANOVA) was applied to the two groups (SG and CG) for three tasks (diagonal, horizontal and vertical), with repeated measures for the last factor (ANOVA-MR), and the following variables were analyzed: average of distance traveled, average time, number of correct answers and number of errors. The *Le*ast Significant Difference (LSD) test was used to detect possible differences. The level of significance adopted was 5%.

## RESULTS

The mean ages of the sample are shown in Table 1, and there was no difference found between groups (p>0.05). As for sex, 26 individuals were female and 28 male. Eleven children were studying in elementary school, nine in middle school, and 14 in high school. SG individuals had, on average, four years and four months (minimum of one year and maximum of ten years) of time instituionalized in shelters.

**Table 1:** Characteristics of the research participants for the assessment of psychomotor battery according to the groups.

Variables	SG (mean ± SD)	CG (mean ± SD)	p-value (mean difference - 95%CI)
Age (in months)	143.7±42.9	143.6±42.9	0.992 (0.1; -23.32-23.54)
Psychomotor battery			
Tonicity	3.5±0.4	3.7±0.3	0.041* (-0.20; -0.39- -0.01)
Balance	3.6±0.4	3.8±0.3	0.163 (-0.13; -0.31-0.05)
Laterality	3.7±0.5	3.8±0.4	0.349 (-0.11; -0.35-0.13)
Body awareness	3.5±0.6	3.7±0.3	0.039* (-0.27; -0.53- -0.01)
Timeline structure	3.2±0.6	3.3±0.5	0.775 (-0.04; -0.34-0.26)
Global apraxia	3.1±0.7	3.1±0.6	0.612 (-0.09; -0.44-0.26)
Fine apraxia	3.2±0.7	3.4±0.5	0.180 (-0.24; -0.59-0.11)
Total	23.4±2.4	24.8±1.8	0.024* (-1.34; -2.50- -0.18)

SG: Sheltered Group; CG: Control Group; SD: standard deviation; 95%CI: 95% confidence interval; *p <0.05.

The findings of PD evaluations by PMB, performed for both groups, are represented in Table 1, with children and adolescents in the SG showing a significant difference in the total score of the instrument (p = 0.024) and in two factors of PMB: tonicity (p = 0.041) and body awareness (p=0.039).

**Figure 2:**
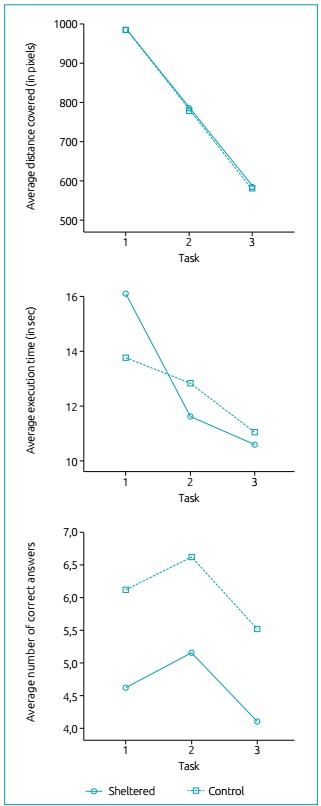
Individuals’ behavior during the tasks, expressed as an average of the distance covered, average execution time and number of correct answers for the three tasks proposed in the Motor Control Assessment software.

The results of the ANOVA-MR for the variables considered for each Task and Group are presented below. The distances covered (Figure 2) were decreasing for the task variable [F (2.104)=2041.3; p <0.001; η^2^= 0.98], and the largest distance was for Task 1 (M = 984.9 pixels) when compared to Tasks 2 (M=783.5 *pixels*) and 3 (M = 583.7 *pixels*). There was no difference in the distance traveled between the groups, regardless of the Task [F (1.52) = 0.2; p = 0.64; η^2^= 0.00].

The average execution time (Figure 2) was different for the Task variable [F(2.104)=21.7; p<0.001; η^2^=0.29]. The post-hoc tests showed that the average time to perform Task 1 (M= 14.9 seconds) was longer when compared to Tasks 2 (M=12.2 seconds) and 3 (M=10.8 seconds). There was no difference in execution time between the groups for all tasks.

Interaction was observed in the mean time for Task and Group [F(2.104)=4.4; p<0.05; η^2^= 0.08]. These differences occurred with the group represented by the participants from the Shelter, with an average time of Task 1 (M = 16.1 seconds) greater than that of Task 2 (M = 11.6 seconds), and also when compared to the time of Task 3 (M = 10.6 seconds), which, however, was only marginally different between Tasks 2 and 3 (p=0.055). When the average CG time was observed, the execution of Task 3 (M = 11.1 seconds) was shorter compared to Tasks 2 (M=12.8 seconds) and 1 (M=13.7 seconds), but there was no difference between Tasks 1 and 2 (Table 2).

**Table 2:** Performance result in the three tasks performed with the Evaluation and Motor Control software, according by groups.

		SG		CG		p-value
		M	SD	M	SD	
Task 1 -Diagonal (Actual distance of 930,053 pixels)	Distance covered (pixels)	984.3	55.1	985.5	46.0	0.93
	Time (Seconds)	16.1	8.1	13.7	4.1	0.19
	Correct answers (n)	4.6	1.6	6.1	2.2	<0.05
	Errors (n)	5.4	1.6	3.9	2.2	<0.05
Task 2 - Horizontal (Actual distance of 750 pixels )	Distance covered (pixels)	787.2	30.9	779.7	21.2	0.30
	Time (Seconds)	11.6	4.4	12.8	5.6	0.38
	Correct answers (n)	5.2	1.8	6.6	2.0	<0.05
	Errors (n)	4.8	1.8	3.4	2.0	<0.05
Task 3 - Vertical (Actual distance of 550 pixels )	Distance covered (pixels)	585.4	26.3	582.1	25.4	0.65
	Time (Seconds)	10.6	3.4	11.1	5.0	0.71
	Correct answers (n)	4.1	1.9	5.5	2.2	<0.05
	Errors (n)	5.9	1.9	4.5	2.2	<0.05

SG: Sheltered Group; CG: Group control; M: mean; SD: standard deviation; n: number.

The number of correct answers was significantly different for the Task variable [F(2.104)=7.1; p=0.001; η^2^=0.12] (Figure 2). The post-hoc tests showed that the CG had more correct answers (M=6.1) compared to SG (M = 4.6), for all tasks, with p <0.05.

## DISCUSSION

This study was interested in analyzing the fine motor control of children and adolescents institutionalized in shelters, and comparing it with individuals who live with their parents. Children and adolescents were subjected to a task that analyzes fine motor control using a computer software, which verifies the distance traveled, the time of movement, the number of errors and correct answers, as performed in another study with hemiparetic patients, in which it was concluded that the respective software’s evaluation was reliable.^14^ ^,^ ^15^ ^,^ ^16^

Fine motor development and, consequently, fine motor control start developing in early childhood and improve during growth, according to the environmental stimuli received. As such, the family nucleus is of extreme importance for such development.^17^ When a child is not inside a well-structured nucleus, there may be a delay in fine motor control. The institutionalization process can be a factor that leads to this delay, since the high number of children living in shelters and the low number of caregivers may not provide those who live there with adequate stimuli for their development.^18^ This is an important justification for analyzing the motor control of individuals in a welcoming situation.

Thus, it was found that, in the evaluation of PD, individuals in shelters had lower MPB scores, with a significant difference, compared to individuals living in a family environment (mean CG=24.8 points; mean SG=23.4 points), which indicates some developmental deficiency.^19^ However, the PD of both groups was still classified as “good” and, therefore, is an age-appropriate development. However, there was a significant difference between the groups in the area of tonicity and body awareness (Tonicity: average CG=3.7 points and average SG=3.5 points; Body awareness: average GC=3.7 points and SG average=3.5 points), in which the individuals in the shelter had greater difficulty in performing the tasks.

Leite et al.^20^ observed that children at school present a normal tone of motor development, however, in some subtasks of the tone test, they obtained low scores, which may result directly from changes in muscle tone, for example, the presence of muscle flaccidity. In this study, however, with the assessment of individuals by PMB, a lower score was found in the area of tonicity in the SG, even though it was considered adequate with regard to the total score of the instrument (which was classified as a good psychomotor profile). This finding indicates that individuals in SG may have a change in muscle tone, which allows for lower scores on the test.

In the study by Coelho,^21^ which also evaluated PD with PMB in individuals with autism spectrum disorder, compared with typical individuals, it was shown that body awareness can be affected by the individual’s anxiety or nervousness.^21^ The shelter children, when subjected to an evaluation, became anxious when starting the evaluations, which could justify the difference in the result of the body awareness test.

For Task 1 - a diagonal line (Figure 1), the longest distance covered for both groups was observed, but there was no difference in the distance covered in all of the tasks between the groups. This fact can be justified by the fact that the children remained focused in order to complete the objective of the task and did not present any complications that could cause them to bring the pen off-course and increase the distance.^22^

The SG took more time to accomplish Task 1 (diagonal line), when compared to Tasks 2 (horizontal line) and 3 (vertical line) (Figure 1) and when compared to the CG findings. The first task performed required more movement time as it had the longest distance between the three straight lines. Additionally, it required the individual to adapt to the use of the pen, the tablet and the software, as it was the first task to be carried out in the evaluation. It can also be highlighted that, because the task was to make a diagonal line, it became more complex when compared to the others, requiring greater concentration and greater skill from the participants.^15^ In the CG, there was no difference between Tasks 1 and 2, but their time on these was longer than on that of Task 3. In addition, it was observed that the CG had greater agility in performing the tasks. This data can be a result of the complexity of the task, the interaction of the individual and the individual’s practice using a computer (non-immersive virtual reality).^9^ Because the task used in this study was non-immersive virtual reality, generally, individuals who have more contact with technology have an easier time performing the activity. Children and adolescents, who were institutionalized in shelters generally have less frequent contact with technology, due to the lack of availability of this equipment. For example, it is only available during classes or during restricted hours in shelters, a fact that can interfere with their manual motor skills. Taking this into consideration, the importance for development of using non-immersive virtual reality equipment (such as cell phones and computers) in an individual’s daily life has already been mentioned in another study ^23^, which may justify the SG findings found in this study.

The number of correct answers during the task attempts was significantly different between the groups in this study, that is, individuals in shelters had a lower number of correct answers than the CG. This demonstrates that the fine motor control of individuals in a host situation may be compromised. The tasks used in this study required total dexterity of hand movements, combined with vision. The lack of early stimuli, such as writing, cutting, and gripping, can cause children to have a deficit in the development of fine motor control.^22^ In addition, Torquato et al.^17^ describe that family contact and interaction provide the necessary stimuli for individuals in the process of development and, as in the present study, individuals in shelters (SG) showed a difference in the scores from the instrument that evaluates PD in relation to those who live in a family environment.

Some limitations of the study included the lack of evaluation of other factors that could influence motor control, such as upper limb strength and hand grip. It was also not possible to record the number of hours per day that individuals in shelters are in touch with technology. However, they do have computer classes and some teenagers have electronic devices (cell phones, tablets etc.), as reported.

The present study points out the need for attention to fine motor control of individuals institutionalized in shelters due to the observed motor control deficit. It is essential to provide this population with a variety of stimuli for their proper development, as the lack of these stimuli can reflect on the future of the individual. This fact determines the need for the daily inclusion of tasks that stimulate the development and, consequently, the motor control of individuals. Therefore, it is concluded that individuals institutionalized in a shelter presented PD below the CG, even though the classification of their development was determined to be good. The sheltered children in this study showed greater difficulty in fine motor control in tasks that required the control of fine and delicate movements of the hands and fingers. When compared to the children in the CG, the children in shelters covered a greater distance in the task that required diagonal movement, took more time in the execution of tasks, and got less correct points and more errors in their performance.
